# Predicting the safety and efficacy of buffer therapy to raise tumour pHe: an integrative modelling study

**DOI:** 10.1038/bjc.2012.58

**Published:** 2012-03-01

**Authors:** N K Martin, I F Robey, E A Gaffney, R J Gillies, R A Gatenby, P K Maini

**Affiliations:** 1 Centre for Mathematical Biology, Mathematical Institute, Oxford University, 24-29 St Giles’, Oxford OX1 3LB, UK; 2 Department of Social Medicine, University of Bristol, Canynge Hall, 29 Whatley Road, Bristol BS8 2PS, UK; 3 University of Arizona, Tucson, AZ, USA; 4 H. Lee Moffitt Cancer Center and Research Institute, 12902 Magnolia Drive, Tampa, FL 33162, USA; 5 Oxford Centre for Integrative Systems Biology, Department of Biochemistry, South Parks Road, Oxford OX1 3QU, UK

**Keywords:** sodium bicarbonate, acidity, mathematical model

## Abstract

**Background:**

Clinical positron emission tomography imaging has demonstrated the vast majority of human cancers exhibit significantly increased glucose metabolism when compared with adjacent normal tissue, resulting in an acidic tumour microenvironment. Recent studies demonstrated reducing this acidity through systemic buffers significantly inhibits development and growth of metastases in mouse xenografts.

**Methods:**

We apply and extend a previously developed mathematical model of blood and tumour buffering to examine the impact of oral administration of bicarbonate buffer in mice, and the potential impact in humans. We recapitulate the experimentally observed tumour pHe effect of buffer therapy, testing a model prediction *in vivo* in mice. We parameterise the model to humans to determine the translational safety and efficacy, and predict patient subgroups who could have enhanced treatment response, and the most promising combination or alternative buffer therapies.

**Results:**

The model predicts a previously unseen potentially dangerous elevation in blood pHe resulting from bicarbonate therapy in mice, which is confirmed by our *in vivo* experiments. Simulations predict limited efficacy of bicarbonate, especially in humans with more aggressive cancers. We predict buffer therapy would be most effectual: in elderly patients or individuals with renal impairments; in combination with proton production inhibitors (such as dichloroacetate), renal glomular filtration rate inhibitors (such as non-steroidal anti-inflammatory drugs and angiotensin-converting enzyme inhibitors), or with an alternative buffer reagent possessing an optimal pK of 7.1–7.2.

**Conclusion:**

Our mathematical model confirms bicarbonate acts as an effective agent to raise tumour pHe, but potentially induces metabolic alkalosis at the high doses necessary for tumour pHe normalisation. We predict use in elderly patients or in combination with proton production inhibitors or buffers with a pK of 7.1–7.2 is most promising.

Malignant tumours generally exhibit upregulated glycolysis and conversion to lactic acid as a result of regional hypoxia caused by disordered blood flow and aerobic glycolysis, in which anaerobic metabolism of glucose persists even in the presence of oxygen – a phenomenon termed the ‘Warburg effect’ (Warburg, 1930). Increased glucose consumption is measured by clinical imaging through positron emission tomography (PET) scanning with ^18^F-deoxy-D-glucose. In fact, extensive clinical experience in imaging human cancers through ^18^F-deoxy-D-glucose PET has demonstrated the vast majority of human malignancies exhibit increased glycolysis (Gambhir, 2002). An inevitable consequence of upregulated glycolysis is production of increased lactic acid. As a result, studies investigating the microenvironment of tumours have demonstrated a consistently low extracellular pH (pHe) – typically in the range of 6.5–7.0 (Helmlinger *et al*, 1997; Schornack and Gillies, 2003). Mathematical modelling of the tumour–host interface (Gatenby and Gawlinski, 1996; Smallbone *et al*, 2007) has predicted that intra-tumoural acidity results in proton flow along concentration gradients from the tumour into the surrounding tissue. This phenomenon has now been confirmed experimentally (Gatenby *et al*, 2006).

Despite the discovery of the Warburg effect nearly 100 years ago, the reason malignant tumours consistently use aerobic glycolysis has remained speculative. The advantage to the tumour cell is not obvious, as anaerobic metabolism of glucose produces only 2 ATP per glucose, as compared with 38 ATP per glucose in aerobic respiration. In addition, glycolysis produces hydrogen ions, which acidify the extracellular space. Why then, would tumours preferentially use glycolysis in well-oxygenated environments?

In previous publications, the authors suggested the glycolytic phenotype confers an evolutionary advantage for tumour cells by producing a harsh acidic environment in adjacent normal tissue, facilitating tumour proliferation and invasion by promoting normal cell death and matrix degradation (Gatenby and Gillies, 2004; Gatenby and Gillies, 2008; Gillies *et al*, 2008). The ‘acid-mediated invasion hypothesis’ leads to the corollary prediction that neutralising acidic tumour pHe will inhibit invasion and, subsequently, spontaneous metastases. Furthermore, such intervention strategies might be achieved with very small molecular buffers such as bicarbonate, which are much more diffusive than drug macromolecules, suggesting prospective treatments that may not suffer from the inhibitory effects of raised interstitial pressures on cross-capillary advective transport within tumours.

Metastasis inhibition through tumour buffering has been confirmed experimentally through oral administration of sodium bicarbonate (NaHCO_3_, pKa 6.3) to severe combined immunodeficient mice bearing MDA-MB-231 and PC3M tumours (Robey *et al*, 2009). However, oral bicarbonate therapy only raised tumour pHe modestly, achieving an increase of ∼0.1 pH unit, and did not inhibit growth of larger, primary tumours nor did it reduce metastases in highly aggressive, rapidly growing cell lines. Further, the pKa of bicarbonate in solution is 6.1, suggesting bicarbonate is a less effective buffer than a buffer with a pKa nearer to blood pH of 7.4.

The current work addresses two issues: (1) Can the experimental bicarbonate therapy results be safely and effectively translated into clinical application as a therapeutic intervention against malignant cancer? (2) Can tumour pHe normalisation be enhanced or more safely delivered through strategies that increase buffer concentration, improve buffering capacity, decrease H^+^ production or excretion, or use buffers of a more optimal pKa? We use a mathematical model of blood and tumour buffering (Martin *et al*, 2011) to recapitulate the previous experimental results in mice bearing metastatic MDA-MB-231 breast tumour xenografts, verifying our model predictions against observed pHe measurements taken using fluorescence ratio imaging of SNARF-1 in a dorsal skin-fold window chamber. We then use *in vivo* studies to test a key model prediction, and predict the translational efficacy in humans. Our modelling predicts effective clinical treatments can be achieved using combination therapies, suggesting promising avenues for new discoveries.

## Materials and Methods

### Mathematical model

**Figure 1:**
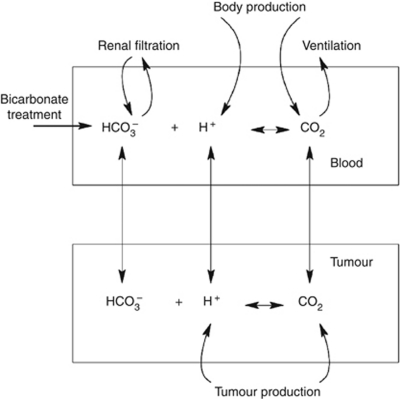
Schematic for the mathematical model. The model tracks concentrations of carbon dioxide, protons and bicarbonate in the blood and tumour compartments. Renal filtration regulates blood levels of bicarbonate through glomerular filtration and acid secretion. The blood receives a constant input of protons and carbon dioxide from the normal tissues. Excess carbon dioxide in the blood is lost through ventilation. The tumour produces acid and carbon dioxide, and all ions can enter and exit the tumour tissue via the tumour vasculature. Reproduced with permission from Martin *et al* (2011).

To examine the effect of buffer administration on blood and tumour pHe, we apply and draw clinical insights from a previously developed simple, but realistic mathematical model of the CO_2_/HCO_3_^−^ buffer system present in blood and tissues. In this analysis, we examine the impact of administration of bicarbonate on blood and tumour pHe in mice and humans. A schematic of the model is shown in Figure 1, details of the model and model verification are presented in the Supplementary Appendix, and a full mathematical asymptotic analysis examining the fast, medium and steady-state dynamics can be found in Martin *et al* (2011).

We use a two-compartment model, representing, respectively, the arterial blood and tumour tissue with a diffusively dominated transport coupling given the small molecules under consideration (consistent with the conclusions that small hydrophilic molecular transport is diffusion dominated in the special case of brain tumours (Groothuis *et al*, 1982; Jain, 1987)). The aim is to develop a simple but realistic model of pHe at the tumour and blood compartment scale, which accurately models the physiological regulation of tumour and blood pHe. The compartments are represented through coupled ordinary differential equations, which neglect spatial variations. The rationale for this model is predicated on blood being well mixed, and that we are interested in the average tumour pHe, rather than smaller scale spatial heterogeneities. As we ignore spatial gradients, we use an averaged proton production term across the tumour. This allows us to neglect the role of heterogeneity in oxygen consumption, especially as tumour cells exhibit the glycolytic phenotype even in the presence of oxygen.

### Numerical simulations and parameterisation

The system of equations is solved in MATLAB using the stiff solver ode 15s, a variable order multistep solver. Initial conditions are normal arterial biological values of the variables (pCO_2_=40 mm Hg, HCO_3_^−^=24 mmol l^−1^, and pHe=7.4). Mouse and human parameters used for the simulations are listed in Supplementary Appendix Table 1. Physiological parameters (such as those involved with ventilation and kidney filtration) and kinetic parameters (such as reaction coefficients) are derived from experimental literature. The tumour acid-production rate is determined by requiring a baseline tumour pHe of 7.0 as found in Robey *et al* (2009). For more details on parameterisation, see Martin *et al* (2011).

### Model verification with bicarbonate administration in mice

To verify whether the model accurately predicts tumour pHe with bicarbonate therapy, we estimate the tumour pHe with the bicarbonate dose administered in the Robey *et al* (2009) study of 36 mmol kg^−1^ per day (an average of 4.2 ml per day per mouse consumption of 200 mM bicarbonate water, and average mouse weight of 23 g). Model predictions were compared with the experimentally observed pHe, which was monitored using fluorescence ratio imaging of SNARF-1 in the dorsal skin-fold window chamber tumour xenografts (Robey *et al*, 2009).

### Model prediction of equivalent dose administration in humans

To examine whether the buffering impact seen in the Robey *et al* (2009) study in mice would be achievable with the same equivalent dose in humans, we simulate the buffer therapy with human parameters and translate the bicarbonate dose. Dose translation from mice to humans is calculated from the Du Bois height–weight formula to predict surface area: BSA (m^2^)=0.007184 × height (cm)^0.725^ × weight (kg)^0.425^ (Freireich *et al*, 1966; Reagan-Shaw *et al*, 2008).

### Animal experiments to determine whether systemic pHe is elevated in bicarbonate-treated mice

The effect of bicarbonate on blood pHe was indirectly measured by sampling urine pHe in tumour-bearing mice. It is well established that a rise in blood pHe because of bicarbonate loading causes a proportionally larger rise in urine pHe (Pitts and Lotspeich, 1946; DuBose, 1982; Nijenhuis *et al*, 2006). Hence, small changes in blood pHe can be monitored via the urine pHe. All animals were maintained under Institutional Animal Care and Use Committee approved protocols at the University of Arizona. In accordance with previous investigations (Robey *et al*, 2009), six- to eight-week-old female severe combined immunodeficient mice (*n*=20) received orthotopic injections of 5 × 10^6^ MDA-MB-231 tumour cells in a mammary fatpad. Animals were monitored and maintained by the Experimental Mouse Shared Services core facility of the Arizona Cancer Center, Tucson Arizona. Six days after injection, mice were randomised into two groups (*n*=10 in each group): the control was provided drinking water and the treated was provided sodium bicarbonate (200 mmol l^−1^) ad libitum, which continued for the duration of the experiment (3 weeks). At intervals, mice from both groups were removed for collection of urine specimens to obtain pH measurements. This was achieved by applying gentle pressure to the abdomen over a plastic membrane.

### Statistics

Statistical calculations were determined using the analysis feature in GraphPad Prism version 4.03. for Windows (GraphPad Software, San Diego CA, USA, http://www.graphpad.com). Unpaired, two-tailed *t*-tests were used to determine if means of urine pHe (found experimentally as described above) at each sample time-point (0, 0.25, 1, 2, 5, 7, 12 and 21 days after treatment initiation) were significantly different between untreated and treated groups. A *P*-value of <0.05 was considered to be statistically significant. Data are represented as mean±s.e.m.

### Model extension to predict the ideal pKa for an alternative buffer

To explore the efficacy and characteristics of an alternative hypothetical buffer (which would ideally have a greater tumour buffering impact and result in less blood pHe change), we develop an extended mathematical model, incorporating the administration and reaction kinetics of a hypothetical exogenously administered buffer. The equations and parameters for this system are detailed in the Supplementary Appendix.

### Assessing clinical implications of model sensitivity

To determine promising combination therapies and potential patient groups who would benefit from treatment, we analyse results of a one-way sensitivity analysis (Martin *et al*, 2011), and perform further simulations to predict clinical impact in humans.

## Results

### Simulations results: bicarbonate raises both tumour and blood pHe, and the model correctly simulates the mouse tumour pHe increase found experimentally

**Figure 2:**
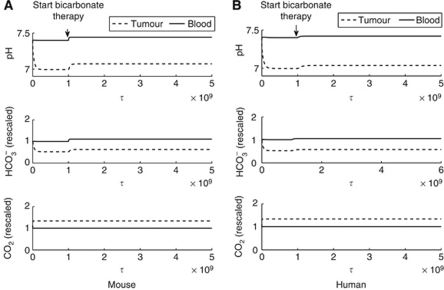
Simulated bicarbonate therapy in a mouse and human over time. The dimensionless time unit *τ* is converted from time, *t,* in seconds, such that *τ*=2.73 × 10^4^*t*, hence, 1 × 10^9^ *τ* equals ∼10 h. (**A**) Mouse: administration of a bicarbonate dose of 36 mmol kg^−1^ day (as in Robey *et al*, 2009) predicts a rise in tumour pHe from 7.0 to 7.07, consistent with window chamber experiments. The blood pH is concurrently elevated from 7.4 to 7.44. Blood bicarbonate levels are normal before treatment initiation (1 denoting the normal state), and elevated after treatment. Tumour bicarbonate levels are low before, and after initiating, treatment (about 50–60% normal levels). Tumour carbon dioxide levels are elevated (about 1.35 times normal), and blood carbon dioxide is maintained as normal throughout treatment. (**B**) Human: using the translated bicarbonate dose (12.5 g per day in a 70 kg human), the simulated human tumour pH is raised from 7.0 to 7.04. The blood pH is elevated to 7.42 from 7.40. As in mice, blood bicarbonate levels are normal before treatment initiation (1 denoting the normal state), and slightly elevated after treatment. Tumour bicarbonate levels are low before, and after initiating, treatment (about 50% normal levels). Tumour carbon dioxide levels are elevated (about 1.35 times normal), and blood carbon dioxide is maintained as normal throughout treatment.

**Figure 3:**
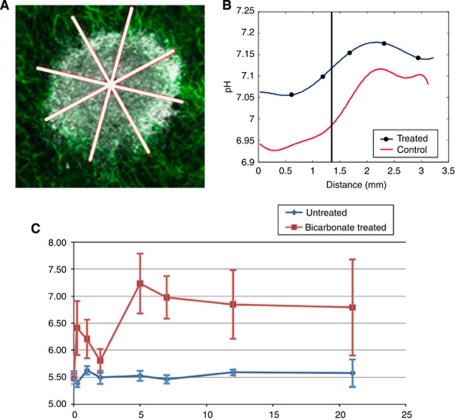
Experimentally derived tumour and urine pHe measurements for bicarbonate-treated and control mice. (**A**) Imaging of the window chamber showing the tumour with abundant vascularisation. The pH measurements were taken along radial lines indicated in white. (**B**) Window chamber least-squares fit across all directions and with all tumours showing pHe distributions along radial lines for control untreated and bicarbonate-treated tumours. The horizontal axis represents the radial distance from the core of the tumour, with the tumour rim indicated by the vertical black line at ∼1.4 mm. Note the raised tumour pH in the buffered mouse and the acidic pH in the interior of the tumour, with a gradient leading out into the normal tissue. The mean (s.e.) pH is 7.0 (0.04) and 7.07 (0.03) for the untreated and treated tumours, respectively (reported in Robey *et al* (2009)). The pH is determined using fluorescence ratio imaging of SNARF-1 as described in Robey *et al* (2009). (**C**) Average urine pHe (vertical axis) for untreated (blue circles) and bicarbonate-treated (red squares) mice through time measured in days (horizontal axis). Measurements were taken at just before treatment (indicated as day 0) and then 6 h after treatment initiation (day 0.25), which already showed a significant rise in urine pHe for the treated mice. Bicarbonate-treated mice show a consistently elevated urine pHe. The mean pHe is shown (circle/square) with the mean±standard error indicated by a bar and vertical line. Figures (**A** and **B**) are adapted with permission from Cancer Research (Robey *et al*, 2009). The colour reproduction of this figure is available at the *British Journal of Cancer* online.

In mouse and human simulations, administration of bicarbonate raises both the tumour and blood pHe (Figure 2). Using the estimated consumed dose of 36 mmol kg^−1^ per day from Robey *et al* (2009), simulations predict an increase of 0.07 pH units in the mouse tumour (from 7.0 to 7.07). This agrees with the observed pHe change recorded using imaging of SNARF-1 in a dorsal skin-fold window chamber, with a mean (s.e.) pHe of the peri-tumoural tissue of 7.0 (0.04) in the control group, and 7.07 (0.03) in the treated group (Figures 3A and B). However, simulations predict bicarbonate raises blood pHe by a smaller relative magnitude (0.04 and 0.07 pH units in mouse blood and tumour, respectively; 0.02 and 0.04 pH units in human blood and tumour, respectively).

### Experimental results: oral administration of bicarbonate consistently elevates urine pHe in mice

Before initiation of bicarbonate therapy, the urine pHe was not statistically different between each group (*P*=0.3). The elevated urine pHe in the bicarbonate treatment group was observable at the first data point after treatment initiation (6 h, *P*<0.001 as compared with the untreated group at *t*=6 h, *P*<0.001 as compared with treatment group at *t*=0). As shown in Figure 3C, the urine pHe in the treatment group as compared with the untreated group remained significantly elevated at each sample time-point through the duration of the study (21 days; *P*<0.001 at hour 6, *P*=0.02 at day 1, *P*=0.01 at day 2, *P*<0.001 at day 5, *P*<0.001 at day 7, *P*<0.001 at day 12, *P*=0.04 at day 21). Similarly, the urine pHe of the treated group remained significantly (*P*<0.01) elevated at each sample time-point as compared with before treatment initiation (at *t*=0).

The relationship between urine and blood pHe is approximately linear in the range studied, but can vary depending on levels of potassium and other ions not measured in this study. Assuming normal ion levels, an estimate for the urine–blood pHe slope can be obtained from a previous bicarbonate-loading study in mice (Nijenhuis *et al*, 2006). Using this slope and assuming a normal, untreated baseline blood pHe of 7.4, the treated urine pHe measurements correspond to an average estimated blood pHe of 7.44±0.02.

### Simulation results: increased bicarbonate dose will be necessary in humans to achieve equivalent tumour pHe elevation, but careful dose monitoring is necessary due to potential blood pHe deregulation

**Figure 4:**
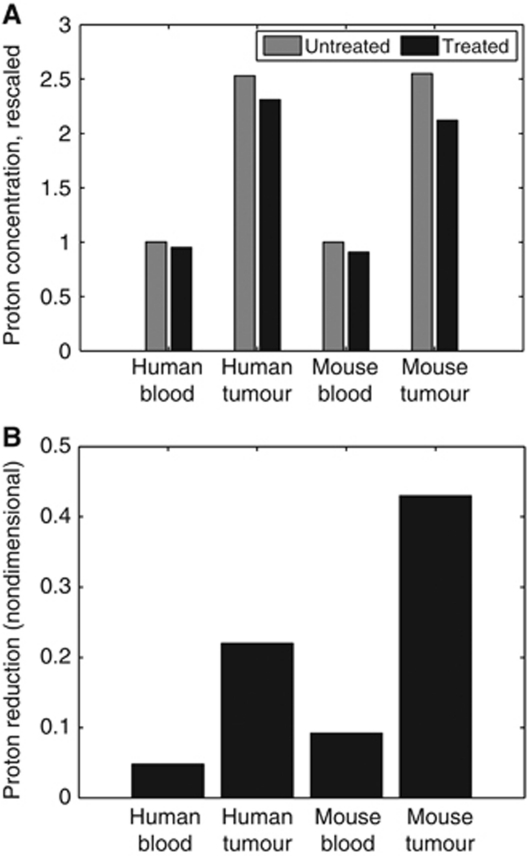
Treatment effect in human *vs* mice in blood and tumour tissue. (**A**) The absolute proton concentrations (rescaled so that normal values are equal to 1) in a treated (black) and untreated (grey) human and mouse. (**B**) Relative proton reductions with the bicarbonate treatment.

An equivalent dose to mice maintained daily on 200 mM sodium bicarbonate (∼36 mmol kg^−1^ per day) is approximately 12.5–15.0 g per day in a 70 kg human. Simulations indicate the equivalent human bicarbonate dose will have a *substantially lower* quantitative effect than in mice (rising the tumour pHe to 7.04 in humans *vs* 7.07 in a mouse), shown in Figures 2 and 4. This highlights the potential need for an increased dose, alternate buffer or combination therapy.

**Figure 5:**
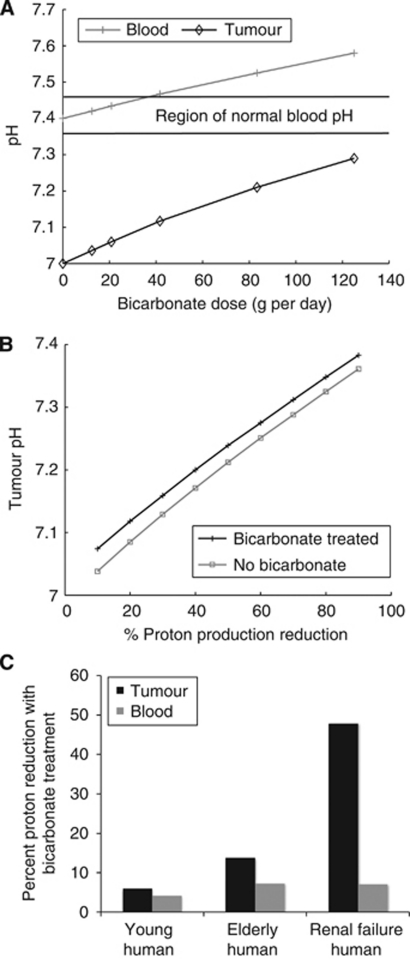
(**A**) Simulated bicarbonate dose response plot in humans, noting the change in pH of the blood (grey crosses) and tumour (black diamonds). The black horizontal lines denote the region of normal blood pH. At a dose of about 35 g of bicarbonate, the predicted tumour pH is elevated to 7.10, with a blood pHe of just below the threshold of systemic alkalosis. Any dose higher than this will potentially elevate the blood pHe into a dangerously high level. (**B**) Predicted effect of proton reduction (where the *x* axis is percent reduction in tumour proton production rate) via a proton inhibitor without bicarbonate (grey squares) or with bicarbonate treatment (black stars). Reducing proton production elevates tumour pHe, but reduces bicarbonate effectiveness. Nevertheless, the net effect of combination therapies that reduce proton production and buffer the tumour could raise the tumour pHe above an ‘acidic threshold’ to promote normal stromal cell function. (**C**) Blood and tumour proton reduction with bicarbonate treatment in young humans (‘young’), elderly patients with mild renal impairment (‘elderly’) and those with chronic renal failure (‘renal failure’). Although the untreated blood is more acidic in patients who are elderly or with chronic renal failure, decreased renal function causes an increased treatment efficacy.

To examine the safety for various doses of chronically administered bicarbonate in humans, we predict dose response curves with respect to blood pHe. As the human body is resilient to altered CO_2_ or bicarbonate levels, we focus primarily on the dangers of altered blood pHe. An optimal treatment would maintain the blood pHe within normal levels (7.35–7.45) but raise tumour pHe to normal levels (7.2–7.4). Predicted dose response plots are shown in Figure 5A.

Metabolic alkalosis is characterised by a blood pHe of above 7.45 (13, 14). In order to maintain the blood pHe below 7.46, the model predicts a maximum dose of 35 g per day of bicarbonate for a 70 kg human, leading to a tumour pHe of 7.10. Maximum daily dose recommendations of oral administration of bicarbonate are around 5 level teaspoons per day, or around 20–25 g per day, and little is known about the safety of long-term chronic ingestion of bicarbonate above this level. The modest predicted tumour pHe change in humans at the maximum safe dose emphasises the importance of using bicarbonate in conjunction with other therapies.

### Simulation results: the optimal pK of an alternative systemic pHe buffer is between 7.1–7.2, but may require higher doses

**Figure 6:**
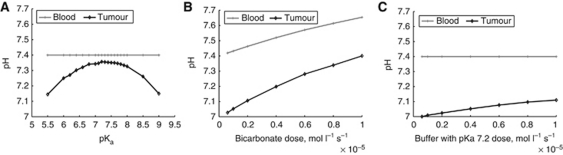
Predicted optimal pKa of a hypothetical buffer and comparative efficacy. (**A**) The optimal pK_a_ is between the pHe of the tumour and tissue. In this case, the optimal pK_a_ is 7.2, directly between the tumour pHe of 7.0 and the blood pHe of 7.4. (**B**) Simulated blood and tumour pHe with bicarbonate of various doses (horizontal axis) in a human. Note the elevation in both blood and tumour pHe with bicarbonate. (**C**) Simulated blood and tumour pHe with a buffer of pK_a_ 7.2 titrated to pHe 7.4. As predicted, the hypothetical buffer titrated to pHe 7.4 does not raise the blood pHe (in contrast to administration of bicarbonate). In both (**B** and **C**), the tumour pHe is elevated, but bicarbonate at the equivalent dose has a larger effect on both blood and tumour pHe.

If the alternative buffer is administered as a solution of acid and conjugate base, titrated to the blood pHe of 7.4, it will increase the buffering capacity of the blood. In contrast to bicarbonate therapy, the model predicts it will not raise the blood pHe, and could hypothetically be given at much higher doses. Simulations indicate the ideal buffer pKa is halfway between the pHe of the tumour and the blood; for a blood pHe of 7.4 and tumour pHe of 6.8–7.0, the optimal buffer pK is 7.1–7.2 (Figure 6A). At equivalent concentrations, bicarbonate is predicted to be more effective at raising tumour pHe than a buffer of pKa 7.2, but bicarbonate elevates blood pHe, unlike the alternate buffer (Figures 6B and C). Hence, higher concentrations of alternative buffers may be required, even at an ideal pKa.

### Simulation results: combination therapy targeting kidney filtration will increase therapy efficacy as well as raise tumour pHe

A sensitivity analysis of the mathematical model indicates kidney filtration terms have the largest effect on tumour pHe (Supplementary Appendix). Consequently, the most effective bicarbonate combination therapy would reduce glomular filtration rate (GFR) or increase the renal acid secretion rate. Targeting these processes would not only raise the tumour pHe, but also increase bicarbonate therapy efficacy due to increased bicarbonate retention.

### Simulation results: elderly patients with renal impairment or renal failure will exhibit an enhanced response to therapy

Based on the sensitivity analysis indicating the importance of renal function on tumour pHe, we examine the potential treatment effect in elderly patients with impaired renal function, and also in patients with chronic renal failure.

As elderly patients traditionally experience a reduction in blood pHe due to nephron death, we simulate bicarbonate administration in a human with impaired renal function. The standard GFR for an elderly patient is 80 ml min^−1^, corresponding to a 35% loss in nephron number and equivalent reduction in acid secretion rate (Hoang *et al*, 2003). With these reductions, the model predicts an increase in therapy efficacy, and a reduction in total tumour pHe (Figure 5C).

Typical chronic renal failure GFR values are 25–35 ml min^−1^, indicating a 75% loss in nephron number and corresponding reduction in acid excretion (Vander, 1980). Simulations with this impairment correctly predict a mild acidosis, with a blood pHe of 7.24, a reduced (70% of normal) bicarbonate level and normal CO_2_ levels, which mirror clinical observations (Singh and Williams, 2008). With the addition of bicarbonate therapy, the model predicts a reduction in blood protons, and an even larger tumour proton reduction (Figure 5C).

### Simulation results: combination therapy targeting tumour proton production (such as dichloroacetate to reduce glycolysis) will selectively raise tumour pHe

The sensitivity analysis also indicates that tumour pHe, but not blood pHe, is sensitive to tumour proton production. Therefore, any agents targeting tumour acid production will selectively lower tumour pHe, with little to no effect on blood pHe. One potential drug that selectively lowers tumour acid production is dichloroacetate. Dichloroacetate (DCA) is an analogue of acetic acid that increases the flux of pyruvate into the mitochondria, promoting glucose oxidation over glycolysis. Experimental studies have shown DCA selectively targets tumour cells, decreasing acid production and inducing apoptosis *in vivo* and *in vitro* (Bonnet *et al*, 2007; Michelakis *et al*, 2008). These two mechanisms serve to decrease overall tumour acidity.

In combination, simulations with bicarbonate and proton inhibition (such as through the administration of DCA) show additive beneficial effects (Figure 5B). Although the proportional efficacy of bicarbonate is lessened with the reduction in proton production by DCA, the net result is an increase in tumour pHe.

## Discussion of mathematical and *in vivo* results

Our modelling results are consistent with experimental observations that bicarbonate acts as an effective agent to raise tumour pHe and there exists significant therapeutic potential of chronic oral bicarbonate therapy. Our study further determines: the translational safety and efficacy in humans; who might benefit the most from bicarbonate therapy; and how buffer therapies might be improved through combination or alternative buffers to decrease intra- and peri-tumoural acidosis. Our model predicts limited impact in humans using equivalent doses of bicarbonate therapy as previously used successfully in mice to prevent metastases. This would be especially true in the case of tumours producing higher concentrations of extracellular acidity; earlier calculations determined that our experimental doses are not saturating and would only be able to counteract the acid production of a 1-mm^3^ tumour (Robey *et al*, 2009).

High bicarbonate doses in humans could substantially raise tumour pHe, but may dangerously elevate blood pHe, thus chronic administration in humans should be closely monitored. Our modelling and *in vivo* experiments are broadly in agreement concerning pHe, predicting roughly the same tumour pHe increase with chronic bicarbonate therapy in mice, and indicate chronic bicarbonate therapy raises blood pHe. Although our previous study (Robey *et al*, 2009) recorded no blood pHe increase, only one measurement was taken at the end of the experiment. Our results conflict with other theoretical models (Silva *et al*, 2009), which predict no change in systemic pHe with bicarbonate administration; however, their model assumes a closed bicarbonate-buffering system in blood, neglecting ventilation and kidney filtration, which are crucial to systemic buffer regulation.

Our simulations predict the use of alternative buffers with a pK of 7.1–7.2 and titrated to a pHe of 7.4 would have negligible effects on blood pHe while reducing tumour acidity. However, modelling indicates these buffers would have to be administered at higher concentrations than bicarbonate to have a similar effect, as ventilation serves to increase bicarbonate-buffering efficacy. A recent study found chronic ingestion of 200 mM solution of a non-volatile buffer, IEPA (pKa 6.9), was effective at reducing metastases in a PC3M mouse model, but resulted in only a marginal increase in tumour pH (Ibrahim Hashim *et al*, 2011). The use of alternative buffers such as IEPA, with a pKa nearer to the optimal range predicted by the modelling, could provide a beneficial rise in tumour pHe without the potential dangers of bicarbonate therapy.

Combination therapy targeting renal filtration could increase therapy efficacy and raise tumour pHe. Some potential drugs that lower GFR are non-steroidal anti-inflammatory drugs (such as aspirin, ibuprofen and naproxen), and angiotensin-converting enzyme inhibitors (commonly prescribed for high blood pressure). As it is highly probable that late middle-aged and elderly patients will already be on these drugs, it is likely that the bicarbonate therapy would have a better effect on them. The use of drugs modifying renal filtration is likely to have significant side effects as well such as electrolyte imbalance, so treatments should be monitored closely. It is also important to note that the blood pHe is exquisitely sensitive to renal function (Supplementary Appendix), and therefore any use of these treatments will cause alterations in blood pHe, which will need to be thoroughly investigated.

Aging and other diseases that affect renal function contribute to higher concentrations of protons in the blood supply. These physiological states would hypothetically be advantageous for users of oral bicarbonate to treat acid-producing cancers because our model indicates they could tolerate higher and more effective doses with lower risk of developing systemic alkalosis. One study reports good tolerance of a chronic high dose (60 g per day) oral administration of sodium bicarbonate in a 79-year-old man with metastatic renal cancer, though blood pHe measurements were not reported (Silva *et al*, 2009). It should be stressed that with the enhancement of bicarbonate therapy under these conditions, patients would likely be subject to other complications or adverse events that should be monitored and investigated thoroughly.

Drugs inhibiting tumour proton production will substantially reduce H^+^ concentrations in tumours. Our model indicates that inhibition of tumour proton production by a relatively small amount could cause substantial reductions in tumour acidity, without any adverse effects on blood pHe. A reagent like DCA fits this criterion because it can reduce extracellular tumour acidity by inhibiting lactic acid production. Moreover, DCA has been shown to selectively target tumour cells (Bonnet *et al*, 2007; Michelakis *et al*, 2008). Our preliminary *in vivo* and *in vitro* experiments using MDA-MB-231 cell lines show that hypoxia could minimise the beneficial impact of DCA as a buffer in this cell type (Robey and Martin, 2011), but other experiments should be performed in cell lines known to respond to DCA, such as A549 and MCF-7. Furthermore, the efficacy of DCA in humans is unclear, and it is important to note that DCA could have possibly serious side effects.

There are a number of limitations of this study. First, the predictions of buffer impact on tumour pHe in humans are based on theoretical modelling predictions and not clinical data. In addition, the mouse dose used in Robey *et al* (2009) study was converted to an equivalent human dose to estimate whether this dose would result in a similar buffering impact. Although translation using allometric inter-species scaling is widely used, it is a crude approach and results should be interpreted cautiously. However, our subsequent estimations of maximally tolerable dose and impact do not rely on this scaling.

Second, our experimental data for the impact of bicarbonate on urine pHe in mice only used one tumour type (MDA-MB-231). The bicarbonate-buffering effect is likely to vary between different tumour types. In our experiments, we use a spontaneously metastatic, highly aggressive tumour model. Therefore, experiments using a less aggressive cell line such as MCF7 could overestimate the potential bicarbonate benefit, but the true effect is unknown as bicarbonate buffering has not been examined in many cell lines. Previous bicarbonate-buffering experiments (Robey *et al*, 2009) also found a bicarbonate effect following tail vein injection of luciferase-expressing PC3M human prostate cancer cells, but no impact using B16 mouse melanoma tumours. Further experiments are needed to determine which cell lines exhibit a response to bicarbonate therapy, and why.

Chronic oral bicarbonate clinical studies have previously been conducted, but not in cancer patients. In these studies, the doses and durations ranged from 12.6 g per day for 46 days to 1.8 g per day for 2 years (de Brito-Ashurst *et al*, 2009; Lemann *et al*, 1965). Clinical trials for oral bicarbonate safety and efficacy in cancer patients are in progress, with the aim to measure dose tolerance and pain intensity over a 4-week-period. A second trial is being planned to assess the feasibility of subjects consuming chronic amounts in a 6-month-period up to the doses discussed in this article.

There is a compelling relationship between the adverse tumour microenvironment and cancer progression. Malignant tumour cells have a critical role in modulating the extracellular environment and there is evidence that this is advantageous for tumour invasion and metastases. The findings presented here show how the administration of exogeneous buffers could modify the tumour microenvironment in humans, specifically in terms of pHe. We elaborate how this process has limitations and may introduce other dangers, but suggest that modest changes in tumour pHe may be sufficient in some cases or helpful in other circumstances to reduce chemoresistance to specific therapies that are stifled by tumour acidity.
