# Supplementary material for: Predicting the safety and efficacy of buffer therapy to raise tumour pHe: an integrative modelling study
Source: Br J Cancer. 2012 Mar 1;106(7):1280–7. doi: 10.1038/bjc.2012.58 (PMC3314784; doi:10.1038/bjc.2012.58)
Supplement: Supplementary Appendix [file bjc201258x1.pdf]

1 Mathematical model

For full details of the mathematical model, please see (Martin et al., 2011). Briefly, the major buffer in the blood and tissues is bicarbonate, which acts via the following chemical reaction that is accelerated by the presence of carbonic anhydrase:

$$HCO_3^- + H^+ \underset{k_2}{\overset{k_1}{\rightleftharpoons}} CO_2 + H_2O. \tag{1}$$

Therefore, our model tracks the levels of bicarbonate, carbon dioxide, and free protons in each compartment. We adjust the reaction rate constants to incorporate the acceleration of the reaction by carbonic anhydrases present in both the blood and tumour. Additional buffering occurs in both blood and tumour compartments due to intracellular buffering, and fixed and mobile protein buffers (Hainsworth, 1986, Davenport, 1974) though these contributions act on a faster timescale than that of the $\mathrm{HCO_3^-}/\mathrm{CO_2}$ buffer. As there is little to no movement of intrinsic buffers between compartments, we assume the buffering contribution in the tumour compartment is constant and implicitly incorporate it in the tumour proton production parameter. The model tracks arterial blood delivery to the tumour, and this blood compartment contains hemoglobin in the oxygen-bound form with low proton carrying capacity. It is reasonable to assume only a small proportion of blood delivered to the tumour (that which is delivered to hypoxic areas) will contain the deoxygenated form of hemoglobin which can bind protons, and hence we neglect this small contribution as a first approximation. The hypoxic tumour subcompartment would be low in bicarbonate, high in $\mathrm{CO_2}$, and likely to have poor flow and connectivity to the vascular network, with the latter which most likely reduces the potential efficacy of any buffer delivery to that region. Subsequently, our model could be extended to incorporate additional buffering components at different spatial and temporal scales. Although it is a first approximation, the model has been validated against known data to ensure accuracy (Robey et al., 2009).

The model is formulated in the blood and tumour compartments, with the chemical reactions, vascular exchanges, physiological responses, and treatment terms. Here, $\mathrm{B}_{t,b}$ represents the bicarbonate in the tumour and blood respectively, $\mathrm{H}_{t,b}$ the free protons, and $\mathrm{C}_{t,b}$ the carbon dioxide.

1.1 The ratio of advection to diffusion in transport across capillaries

To ascertain which transport mechanisms the model should consider, we need to assess the relative importance of diffusion compared to advection for transport across capillaries, which is measured by the Peclet Number, P_e. In the context of microvascular permeability this is given by (Jain, 1987):

$$P_e = \frac{(\text{Hydraulic Conductivity})|\Delta p - \sigma \Delta \Pi|(1-\sigma_F)}{\text{Diffusive Permeability}} \leq \frac{(\text{Hydraulic Conductivity})|\Delta p - \sigma \Delta \Pi|}{\text{Diffusive Permeability}}. \tag{2}$$

Here Δp and $\Delta \Pi$ are the hydrodynamic and osmotic pressure drops across the capillary, with σ and σ_F representing the osmotic and solvent drag reflection coefficients, respectively, which are constrained between zero and unity. Note that we have used the modulus of $\Delta p - \sigma \Delta \Pi$ in the above to ensure that $P_e > 0$, as is typical in the engineering and physical literature.

When P_e is small compared to unity, transport is dominated by diffusion: hence to demonstrate that biophysical measurements indicate the movement of bicarbonate across capillaries is diffusively dominated it is sufficient to consider the upper bound of P_e on the right hand side of inequality (2).

Estimating the above contributions to the Peclet number is difficult due to tissue variation so we consider typical scales for a single tissue to give an indication of its typical size. Bicarbonate vascular diffusive permeability has been measured in frog muscle, yielding a

value of 3.4×10^{-5}cm s^{-1}(Jain, 1987, Olesen and Crone, 1983), with microvascular permeability measurements of essentially the same size (Olesen and Crone, 1983). Frog muscle vascular hydraulic conductivity is of approximately 8 x 10^{-8}cm s^{-1} per cm H_20 (Jain, 1987). The parameter grouping $|\Delta p - \sigma \Delta \Pi|$ varies between 5-10 mm Hg from the arteriolar and the capillary to its venous end for a reflection coefficient $\sigma = 1$ (Brandis, 2011). Reducing the reflection coefficient acts to reduce this variation though the most extreme tumour interstitial pressure measured is 23mm Hg (Jain, 1987), which increases this parameter grouping to 43 mm Hg near the venous end of the capillary. Noting that mercury is approximately 13.6 times more dense than water (1 mmHg=13.6mm H_20=1.36 cm H_20), we have

$$
\begin{aligned}
P_e &= \frac{8 \times 10^{-8}\text{cm s}^{-1}\text{per cm H}_2\text{O} \times 43\text{mm Hg}}{3.4 \times 10^{-5}\text{cm s}^{-1}} \\
&= \frac{1.09 \times 10^{-7}\text{cm s}^{-1}\text{per mm Hg} \times 43\text{mm Hg}}{3.4 \times 10^{-5}\text{cm s}^{-1}} = 0.14 << 1
\end{aligned}
\tag{3}
$$

and hence our parameter estimates indicate that even when considering extreme tumour interstitial pressures, diffusion is the dominant transport mechanism for cross capillary bicarbonate transport for the frog tissue considered. More extensive conclusions could be drawn if further measurements in different tissues for diffusive permeabilities and hydraulic conductivities were available. In particular, vascular permeability is observed to increase in tumours though quantitative measurements have not been made (Jain, 1987); the hydraulic conductivity is also generally anticipated to increase, though no detailed measurements are available (*ibid*). In the absence of further information, we assume any increase in hydraulic conductivity within tumours does not swamp the increase in vessel permeability, which is consistent with the conclusions that small hydrophilic molecule transport is diffusion dominated for the special case of brain tumours (Jain, 1987, Groothuis et al., 1982). As such, to the extent that the data allow conclusions to be drawn, bicarbonate cross capillary transport appears to be diffusion dominated.

In the absence of detailed quantitative information on hydrogen ion permeabilities and noting that small positive ions (Na^+, K^+) have a slightly enhanced diffusive permeability relative to bicarbonate in frog muscle (Jain, 1987), we hypothesise by inheritance that hydrogen ion transport across capillaries is also diffusion dominated, even in tumours.

It is reasonable to assert that carbon dioxide transport across capillaries is diffusively dominated: otherwise extensive regions of the capillary bed could not remove excess carbon dioxide during normal metabolism. Capillary diffusive permeability measurements for carbon dioxide are lacking, though these are anticipated to be roughly the same as those measured for lipid bilayers (Geers and Gros, 2000), which are four orders of magnitude larger than capillary permeabilities of bicarbonate (Jain, 1987, Gutknecht et al., 1971), quantitatively demonstrating that carbon dioxide transport is diffusively dominated.

Consequently all model constituents are taken to be subject to diffusively dominated transport across capillaries and the fluxes coupling the tumour and blood compartments of the model are driven by concentration differences, independently of interstitial pressures, by inheritance from diffusive transport.

1.2 Building the Model: Tumour Compartment.

The acid-base dynamics in the tumour compartment are:

$$\frac{dB_t}{dt} = \overbrace{k_2C_t - k_1B_tH_t}^{\text{chemical reactions}} + \overbrace{\gamma_1(B_b - B_t)}^{\text{vascular exchange}} \tag{4}$$

$$\frac{dH_t}{dt} = \overbrace{k_2C_t - k_1B_tH_t}^{\text{chemical reactions}} + \overbrace{\phi_1}^{\text{tumour production}} - \overbrace{\gamma_2(H_t - H_b)}^{\text{vascular exchange}} \tag{5}$$

$$\frac{dC_t}{dt} = \overbrace{k_1B_tH_t - k_2C_t}^{\text{chemical reactions}} + \overbrace{\phi_5}^{\text{tumour production}} - \overbrace{\gamma_3(C_t - C_b)}^{\text{vascular exchange}}, \tag{6}$$

with all concentrations in mol/L, and initial conditions set as the normal arterial blood values, $C_t(0) = c_0$, $B_t(0) = b_0$, and $H_t(0) = h_0$. Here, γ_1, γ_2, γ_3 are the vessel fluxes for

bicarbonate, lactate, and carbon dioxide respectively. These are given by $\gamma_i = VAD \times P_i$ where VAD is the vessel length per tumour cross section area (in cm/cm^2), and P_i is the vessel permeability (in cm/s) for the respective ion or molecule (Jain, 1987). The ϕ terms are the tumour production terms of protons and carbon dioxide.

We assume that the hydration and dehydration reaction rates (k_1 and k_2) for the HCO_3^-/CO_2 conversion are equal in the blood and the tumour. In the blood, carbonic anhydrase (CA) II in red blood cells can accelerate the hydration reaction 50,000 to 1,000,000 fold over the uncatalyzed rate at human body termperature Chegwidden and Edwards (2000). Tumour associated carbonic anhydrases include CA II and CA IX (Nordfors et al., 2010, Chia et al., 2001); the activity of CA IX has recently been found to be as high as CA II (Hilvo et al., 2008). Hence, we assume for simplicity the catalytic rates in the blood and tumour are equal, though a previous analysis indicates the model is robust to changes of several orders of magnitude in k_1 and k_2, provided the ratio of the reaction rates, and hence pKa, remains equal (Martin et al., 2011).

1.3 Building the Model: Blood Compartment.

The acid-base dynamics in the blood compartment are:

$$\frac{dB_b}{dt} = \overbrace{k_2C_b - k_1B_bH_b}^{\text{chemical reactions}} + \overbrace{\phi_2C_b - \lambda_1B_b}^{\text{kidney filtration}} + \overbrace{\theta_1}^{\text{treatment}} - \overbrace{\gamma_1 v_T(B_b - B_t)}^{\text{vascular exchange}} \tag{7}$$

$$\frac{dH_b}{dt} = \overbrace{k_2C_b - k_1B_bH_b}^{\text{chemical reactions}} + \overbrace{\phi_3}^{\text{body production}} + \overbrace{\gamma_2 v_T(H_t - H_b)}^{\text{vascular exchange}} \tag{8}$$

$$\frac{dC_b}{dt} = \overbrace{k_1B_bH_b - k_2C_b}^{\text{chemical reactions}} + \overbrace{\phi_4}^{\text{body production}} - \overbrace{\lambda_2C_bf(C_b)}^{\text{ventilation}} + \overbrace{\gamma_3 v_T(C_t - C_b)}^{\text{vascular exchange}}. \tag{9}$$

The initial conditions are set as the normal arterial blood values, $C_b(0) = c_0$, $B_b(0) = b_0$ and $H_b(0) = h_0$. In these equations, v_T represents the volume fraction of the tumour/blood,

which varies as the tumour grows, but will be considered constant in these simulations as that is an appropriate assumption over our time span.

The model incorporates renal filtration of blood bicarbonate via the ϕ_2 and λ_1 terms, a detailed explanation can be found in (Martin et al., 2011). The amount of bicarbonate lost from the bloodstream to the kidney is proportional to the blood concentration of bicarbonate and ϕ_2, the glomerular filtration rate (GFR). The GFR is a combined rate of the amount of bicarbonate filtered from the blood in all of the nephrons in the kidney. The rate of bicarbonate absorption is equivalent to the rate of net total acid excretion λ_1, via the splitting of blood CO_2 by intracellular carbonic anhydrase. Although bicarbonate re-absorption in the nephron is a complicated process involving several other ions, this type of mathematical representation is commonly used in calculating acid/base disturbances (Hainsworth, 1986, Davenport, 1974). The terms ϕ_3 and ϕ_4 represent the contribution from the rest of the body tissues into the blood of protons and carbon dioxide, respectively.

1.4 Building the Model: Ventilation.

In the system, we construct a ventilation term where CO_2 lost through ventilation is proportional to the product of the ventilation rate and the CO_2 concentration. The function for ventilation rate is approximately linear with minimum and maximum thresholds (Widdicombe and Davies, 1983). This curve has been well quantified experimentally in both humans and mice (Mitchell and Singer, 1965, Fencl et al., 1969, Yee and Scarpelli, 1986). Essentially, CO_2 lost through ventilation is proportional to the product of the ventilation rate, $f(C_b)$, and the CO_2 concentration. The function for ventilation we use is:

$$f(C_b) = \begin{cases} V_{min} & \text{if } f(C_b) < V_{min}, \\ V_{slope}C_b - V_{intercept} & \text{if } V_{min} < f(C_b) < V_{max}, \\ V_{max} & \text{if } f(C_b) > V_{max}. \end{cases} \tag{10}$$

We neglect the effect of H^+ on ventilation rate as the presence of respiratory compensation to metabolic alkalosis (our examined state) is controversial, often not present in humans and dogs, and when present the magnitude of compensation is highly variable and in all cases limited to a low level (Roberts et al., 1956, Poppell et al., 1956, Javaheri et al., 1982, Hornick, 2003, Feldman and Zimmerman, 2001).

1.5 Nondimensionalisation

We use the rescaling $\tau = k_2 t$, $b_0 b_t = B_t$, $c_0 c_t = C_t$, $h_0 h_t = H_t$, $b_0 b_b = B_b$, $c_0 c_b = C_b$, and $h_0 h_b = H_b$ to nondimensionalise the model, and obtain the system:

$$\frac{db_t}{d\tau} = \delta_1(c_t - \alpha_2 b_t h_t) + \Gamma_1(b_b - b_t) \tag{11}$$
$$\frac{dh_t}{d\tau} = \delta_3(c_t - \alpha_2 b_t h_t) + \Phi_1 - \Gamma_2(h_t - h_b) \tag{12}$$
$$\frac{dc_t}{d\tau} = -(c_t - \alpha_2 b_t h_t) + \Phi_5 - \Gamma_3(c_t - c_b) \tag{13}$$
$$\frac{db_b}{d\tau} = \delta_1(c_b - \alpha_2 b_b h_b) + \Phi_2 c_b - \xi_1 b_b + \Theta_1 - \Gamma_1 v_T(b_b - b_t) \tag{14}$$
$$\frac{dh_b}{d\tau} = \delta_3(c_b - \alpha_2 b_b h_b) + \Phi_3 + \Gamma_2 v_T(h_t - h_b) \tag{15}$$
$$\frac{dc_b}{d\tau} = -(c_b - \alpha_2 b_b h_b) + \Phi_4 - \xi_3(c_b)c_b + \Gamma_3 v_T(c_t - c_b), \tag{16}$$

with $\delta_1 = \frac{c_0}{b_0}$, $\alpha_2 = \frac{k_1 h_0 b_0}{k_2 c_0}$, $\Gamma_1 = \frac{\gamma_1}{k_2}$, $\delta_3 = \frac{c_0}{h_0}$, $\Phi_1 = \frac{\phi_1}{k_2 h_0}$, $\Gamma_2 = \frac{\gamma_2}{k_2}$, $\Gamma_3 = \frac{\gamma_3}{k_2}$, $\Phi_2 = \frac{\phi_2 c_0}{k_2 b_0}$, $\xi_1 = \frac{\lambda_1}{k_2}$, $\Theta_1 = \frac{\theta_1}{k_2 b_0}$, $\Phi_3 = \frac{\phi_3}{k_2 h_0}$, $\Phi_4 = \frac{\phi_4}{k_2 c_0}$, and $\Phi_5 = \frac{\phi_5}{k_2 c_0}$. Additionally, the nondimensionalised

ventilation function is now:

$$\xi_3(c_b) = \begin{cases} \Delta_{min} & \text{if } \xi_3(c_b) < \Delta_{min}, \\ \Delta_1 c_b - \Delta_2 & \text{if } \Delta_{min} < \xi_3(c_b) < \Delta_{max}, \\ \Delta_{max} & \text{if } \xi_3(c_b) > \Delta_{max}, \end{cases} \tag{17}$$

with $\Delta_{min} = \frac{\lambda_2}{k_2} V_{min}$, $\Delta_1 = \frac{\lambda_2}{k_2} V_{slope} c_0$, $\Delta_2 = \frac{\lambda_2}{k_2} V_{intercept}$, and $\Delta_{max} = \frac{\lambda_2}{k_2} V_{max}$.

The initial conditions become:

$$c_b(0) = 1,\ c_t(0) = 1,\ b_b(0) = 1,\ b_t(0) = 1,\ h_b(0) = 1,\ \text{and } h_t(0) = 1. \tag{18}$$

2 Sensitivity analysis

The calculation of a sensitivity coefficient allows the quantification of the effect a change in a parameter, p, has on one of the variables, V. This can be calculated by the equation,

$$S_{V,p} = \frac{p}{V} \frac{\partial V}{\partial p} \tag{19}$$

allowing the identification of parameters which have the most effect in altering the tumour pHe, as well as how the treatment term can affect the pHe of the tumour and blood. The full derivation and results are presented in (Martin et al., 2011), but a subset of these results and clinical implications are discussed in this manuscript.

3 Hypothetical exogenous buffer

An important treatment alternative is the option to use an alternate buffer to sodium bicarbonate, or a combination of bicarbonate and another buffer. Therefore, we extend the

previous model to include an additional non-volatile hypothetical buffer. With this extended model, it is possible to explore the ideal characteristics of a hypothetical buffer, thereby highlighting the buffers with the most treatment potential.

Here we extend our system by modelling the addition of a hypothetical buffer solution which contains the buffer in its conjugate base form, A^-, and its acid, D, which react in the following manner:

$$A^- + H^+ \underset{k_4}{\overset{k_3}{\rightleftharpoons}} D. \tag{20}$$

Both the acid and its conjugate base can diffuse from the blood into the tumour. In the body there are organic and inorganic proteins which act in this way, although they are at such low concentrations that they have only a minimal effect on blood buffering. However, we would like to know if a treatment involving an alternative buffer with a different pK_a would have a better clinical outcome, and if so, what pK_a or buffer to try in future experiments.

As before, $B_{t,b}$ represents the bicarbonate buffer in the tumour and blood respectively, $H_{t,b}$ the free protons, and $C_{t,b}$ the carbon dioxide. In addition, the model includes the hypothetical buffer in its conjugate base form, A, and its acid, D. The model additions are noted by the overbraces and descriptions.

The tumour equations are as follows:

$$\frac{dB_t}{dt} = k_2 C_t - k_1 B_t H_t + \gamma_1 (B_b - B_t) \tag{21}$$

$$\frac{dH_t}{dt} = k_2 C_t - k_1 B_t H_t + \overbrace{k_4 D_t - k_3 A_t H_t}^{\text{non-bicarbonate buffering}} + \phi_1 - \gamma_2 (H_t - H_b) \tag{22}$$

$$\frac{dC_t}{dt} = k_1 B_t H_t - k_2 C_t + \phi_5 - \gamma_3 (C_t - C_b) \tag{23}$$

$$\frac{\overbrace{dA_t}^{\text{buffer base}}}{dt} = \overbrace{k_4 D_t - k_3 A_t H_t}^{\text{reaction kinetics}} + \overbrace{\gamma_4 (A_b - A_t)}^{\text{vascular transfer}} \tag{24}$$

$$\frac{\overbrace{dD_t}^{\text{buffer acid}}}{dt} = \overbrace{k_3 A_t H_t - k_4 D_t}^{\text{reaction kinetics}} - \overbrace{\gamma_5 (D_t - D_b)}^{\text{vascular transfer}}. \tag{25}$$

The blood equations are as follows:

$$\frac{dB_b}{dt} = k_2C_b - k_1B_bH_b + \phi_2C_b - \lambda_1B_b + \theta_1 - \gamma_1 v_T(B_b - B_t) \tag{26}$$

$$\frac{dH_b}{dt} = k_2C_b - k_1B_bH_b + \overbrace{k_4D_b - k_3A_bH_b}^{\text{non-bicarbonate buffering}} + \phi_3 + \gamma_2 v_T(H_t - H_b) \tag{27}$$

$$\frac{dC_b}{dt} = k_1B_bH_b - k_2C_b + \phi_4 - \lambda_2C_bf(C_b) + \gamma_3 v_T(C_t - C_b) \tag{28}$$

$$\overbrace{\frac{dA_b}{dt}}^{\text{buffer base}} = \overbrace{k_4D_b - k_3A_bH_b}^{\text{reaction kinetics}} + \overbrace{\theta_6}^{\text{treatment}} - \overbrace{\lambda_3A_b}^{\text{loss}} - \overbrace{\gamma_4 v_T(A_b - A_t)}^{\text{vascular transfer}} \tag{29}$$

$$\overbrace{\frac{dD_b}{dt}}^{\text{buffer acid}} = \overbrace{k_3A_bH_b - k_4D_b}^{\text{reaction kinetics}} + \overbrace{\theta_7}^{\text{treatment}} - \overbrace{\lambda_4D_b}^{\text{loss}} + \overbrace{\gamma_5 v_T(D_t - D_b)}^{\text{vascular transfer}} \tag{30}$$

with initial conditions,

$$C_b(0) = c_0,\ C_t(0) = c_0,\ B_b(0) = b_0,\ B_t(0) = b_0,\ H_b(0) = h_0,\ H_t(0) = h_0$$
$$D_b(0) = 0,\ D_t(0) = 0,\ A_b(0) = 0,\ A_t(0) = 0. \tag{31}$$

As in the previous model, γ_4 and γ_5 are the vessel flux rates. The treatment terms are θ_6 and θ_7, and λ_3 and λ_4 represent general loss terms, such as due to kidney filtration. The actual physiology of this term may differ depending on the particular buffer, but this general loss term would still be appropriate.

Importantly, we explore two different types of treatment in this section: untitrated and titrated. If the treatment is *untitrated*, then the total dose of buffer (M/L/sec), TotDose, is administered in the A form. Hence, $\theta_6 =$TotDose and $\theta_7 = 0$. If the treatment is *titrated* then H^+ is added to the A form to create a solution of A and D at any desired pH. Therefore, if the treatment is titrated to the blood pH of 7.4, then $\theta_6 = \left(1 - \frac{1}{1+10^{7.4-pK_a}}\right)\times$TotDose, and $\theta_7 = \frac{1}{1+10^{7.4-pK_a}}\times$ TotDose. The advantage of this is that a buffer can be taken with any pK_a, and if it is titrated to the blood pH of 7.4 it will not change the blood pH when

administered as a treatment. Instead, it will just increase the concentration of buffer in the blood, increasing the buffering capacity.

The ventilation term, $f(C_b)$, remains the same as in our original model,

$$f(C_b) = \begin{cases} V_{min} \text{ L/s} & \text{if } f(C_b) < V_{min}, \\ V_{slope}C_b - V_{intercept} \text{ L/s} & \text{if } V_{min} < f(C_b) < V_{max}, \\ V_{max} \text{ L/s} & \text{if } f(C_b) > V_{max}. \end{cases} \tag{32}$$

and the human parameter values used are in Table 1.

We assume that the hypothetical buffer is similar in size to HCO_3^-, and therefore has approximately the same vessel permeability ($\gamma_4 = \gamma_5 = \gamma_1$). In reality, size, charge, and solubility will all affect its delivery to the tumour. As we assume the buffer is completely exogenous, the initial conditions of each are zero. The ratio of k_4 and k_3 is determined by the pK$_a$ we choose. As the specific values of k_3 will differ among buffers, we estimate that the reactions proceed on the same timescale as the bicarbonate reactions, and let $k_1 = k_3$ and from the equation for pK$_a$, calculate $k_4 = k_3 10^{-pK_a}$. The values of θ_6 and θ_7 are determined by our initial dose and titration. If the buffer is solely administered in the A form, then $\theta_7 = 0$. If the solution is titrated to the blood pH, then the total dose is split between the D and A forms. We assume that the buffer is lost through renal filtration in the glomerulus, therefore $\lambda_3 = \lambda_4 = \lambda_1$ as GFR is constant regardless oft he exogenous buffer molecular size. The parameters used are summarised in Table 2, and doses are (θ_6, θ_7) listed in the figures.

Name	Mouse	Human	Units	Source (M: mouse, H: Human)
h_0	3.98×10^{-8}	3.98×10^{-8}	mol/L	M:(Green, 1966, The Jackson Laboratory, 2009) H: (Davenport, 1974)
b_0	2.4×10^{-2}	2.4×10^{-2}	mol/L	M:(Green, 1966, The Jackson Laboratory, 2009) H:(Davenport, 1974)
c_0	1.2×10^{-3}	1.2×10^{-3}	mol/L	M:(Green, 1966, The Jackson Laboratory, 2009) H:(Davenport, 1974)
P_1	3.4×10^{-5}	3.4×10^{-5}	cm/s	(Jain, 1987, 2005)
P_2	1.2×10^{-4}	1.2×10^{-4}	cm/s	(Gatenby et al., 2006)
P_3	1×10^{-3}	1×10^{-3}	cm/s	(Endeward, 2005, Cooper and Boron, 1998)
VAD	20	20	cm/cm^2	(Monsky et al., 2002, Missbach-Guentner et al., 2008, Gee et al., 2003)
v_T	0.1	0.01	$--$	M: (Robey et al., 2009, Dadiani et al., 2004)<br>H:(Singletary et al., 2002, Dadiani et al., 2004)
ϕ_1	7.8×10^{-6}	7.8×10^{-6}	mol/L/s	fit to (Robey et al., 2009), within the range from<br>(Martin and Jain, 1994, Gatenby et al., 2006)
ϕ_4	3.7×10^{-5}	3×10^{-6}	mol/L/s	M:(Mackey and Glass, 1977, Green, 1966, The Jackson Laboratory, 2009)<br>H: (Mackey and Glass, 1977)
ϕ_2	6.16×10^{-2}	1.14×10^{-2}	1/s	M:(Chambrey et al., 2005) H: (Hainsworth, 1986, Davenport, 1974)
ϕ_3	1.5×10^{-6}	1.2×10^{-6}	mol/L/s	M: (Martin and Jain, 1994, Green, 1966)<br>H: (Martin and Jain, 1994, Hainsworth, 1986)
ϕ_5	2×10^{-7}	2.5×10^{-7}	mol/L/s	M: (Mackey and Glass, 1977, Green, 1966)<br>H:(Mackey and Glass, 1977, Hainsworth, 1986)
λ_1	3×10^{-3}	5.2×10^{-4}	1/s	M:(Meneton et al., 2000, Levine et al., 2008)<br>H: (Davenport, 1974, Pitts, 1970, Vander, 1980, Lote, 1999)
λ_2	102	0.042	1/L	M:(Wetterlin and Pettersson, 1979)<br>H: (Hainsworth, 1986, Davenport, 1974)
k_2	2.73×10^4	2.73×10^4	1/s	(from pK_a in (Hainsworth, 1986, Davenport, 1974))
k_1	3.437×10^{10}	3.437×10^{10}	L/mol × s	(Hainsworth, 1986, Putnam and Roos, 1991)
V_{slope}	0.34	1.1×10^3	L^2/mol× s	M:(Yee and Scarpelli, 1986) H:(Mitchell and Singer, 1965, Fencl et al., 1969)
V_{max}	5.5×10^{-4}	1	L/s	M:(Yee and Scarpelli, 1986) H:(Mitchell and Singer, 1965, Fencl et al., 1969)
V_{min}	1.4×10^{-4}	0.02	L/s	M:(Yee and Scarpelli, 1986) H:(Mitchell and Singer, 1965, Fencl et al., 1969)
$V_{intercept}$	9.4×10^{-5}	1.237	L/s	M:(Yee and Scarpelli, 1986) H:(Mitchell and Singer, 1965, Fencl et al., 1969)
θ_1	7.6×10^{-6}	6×10^{-7}	mol/L×s	M: (Robey et al., 2009) H: (Robey et al., 2009, Freireich et al., 1966)

Table 1: Parameters used in the mathematical simulations. The sources are noted, and full derivation of the parameters can be found in Martin et al. (2011).

Name	Value	Units
k_4	$k_3 10^{-pK_a}$	1/s
k_3	k_1	L/mol $\times$s
γ_4	γ_1	1/s
γ_5	γ_1	1/s
λ_3	λ_1	1/s
λ_4	λ_1	1/s

Table 2: Hypothetical buffer parameter values.

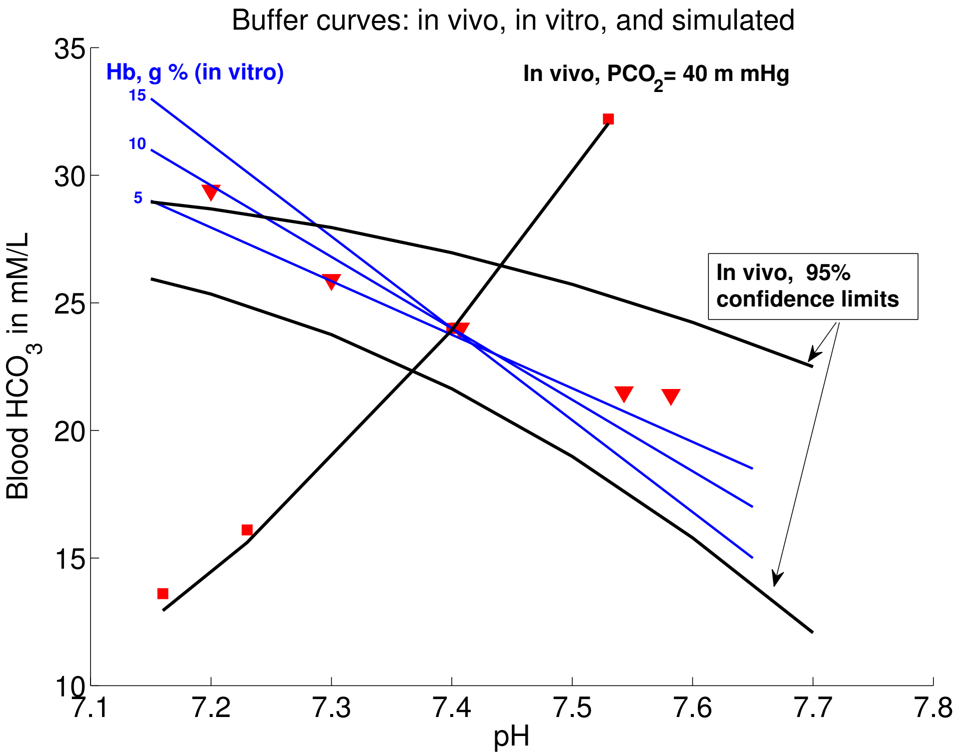

Figure 1: Human buffer curve comparison between *in vitro*, *in vivo*, and calculated with our model. Blue lines represent *in vitro* curves of blood containing varying amounts of haemoglobin. Dark black lines are the *in vivo* observed ranges in values for a normal human. Red triangles and squares indicate calculated values of pH with the induction of metabolic or respiratory disturbances (see (Martin et al., 2011) for full details and parameters). Red squares represent inducing a metabolic disturbance by varying HCO_3^- with a constant pCO_2 level (40 mm Hg). Red triangles represent the effect of varying CO_2 through disordered ventilation rates. These data points were obtained by fixing the ventilation rate at several values, running the simulations and taking the blood CO_2, HCO_3^-, and pH values prior to renal compensation consistent with experiments. Following the pCO2 isopleth, the model produces an excellent fit, and falls within normal limits for the *in vitro* blood buffer line. In comparing the model predictions to the *in vivo* data, the model falls within the 95 % confidence limits of the experimental data, particularly within the biological pH range of primary importance (7.35-7.45). Only at very acidic pH is there a deviation from the predicted buffer line, which is acceptable as the model is primarily focused on the potential creation of metabolic alkalosis, not acidosis. Deviations from *in vitro* and *in vivo* measurements are likely due to electrolyte distribution and different buffering capacities of cells and interstitial fluid. Reproduced from (Martin et al., 2011).

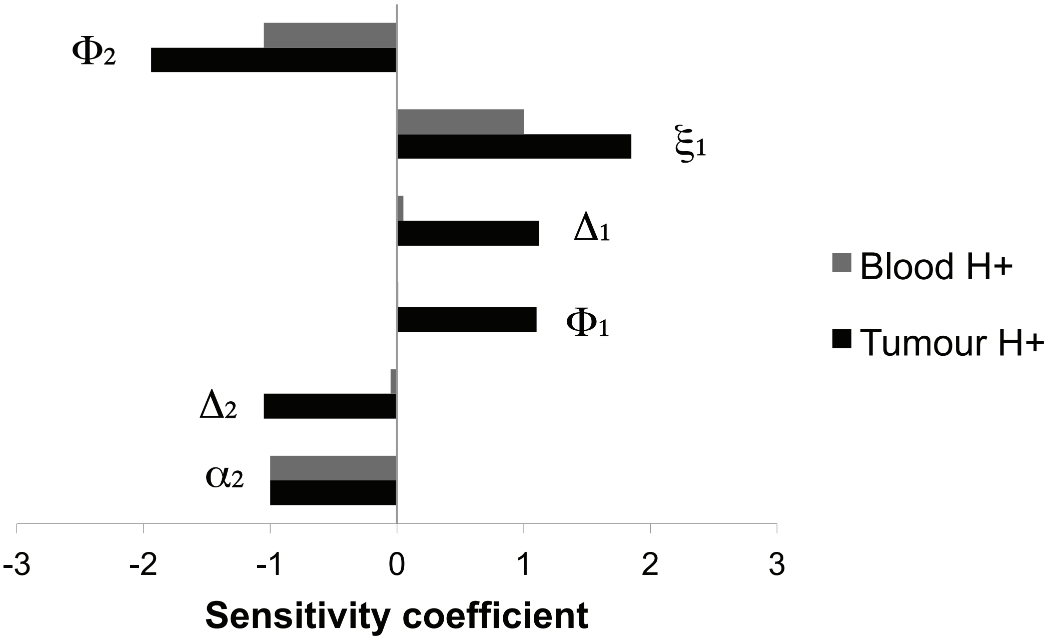

Figure 2: Human sensitivity coefficients with an absolute value greater than unity, with treatment, $\theta_1 = 6 \times 10^{-7}$. The magnitude indicates how sensitive the tumour and blood proton concentrations are to a particular parameter, with larger magnitudes indicating more sensitivity. Notably, the tumour proton level is most sensitive to the parameters involved with renal function Φ_2 and ξ_1, but the blood proton level is also highly sensitive to changes in these as well. Alternatively, the tumour proton concentration is also sensitive to parameters involved with tumour proton production (Φ_1) and ventilation (Δ_1 and Δ_2), and the blood proton level is not as sensitive to changes in these.

References

Brandis, K. (2011). *Fluid Physiology.* http://www.AnaesthesiaMCQ.com/FluidBook/index.php.

Chambrey, R., Goossens, D., Bourgeois, S., Picard, N., Bloch-Faure, M., Leviel, F., Geoffroy, V., Cambillau, M., Colin, Y., Paillard, M., Houillier, P., Cartron, J., and Eladari, D. (2005). Genetic ablation of Rhbg in the mouse does not impair renal ammonium excretion. *American Journal of Physiology – Renal Physiology*, 289(6):F1281–90.

Chegwidden, C. and Edwards, E. (2000). *The Carbonic Anhydrases: New Horizons.* Birkhauser Press.

Chia, S., Wykoff, C., Watson, P., Han, C., Leek, R., Pastorek, J., Gatter, K., Ratcliffe, P., and Harris, A. (2001). Prognostic significance of a novel hypoxia-regulated marker, carbonic anhydrase IX, in invasive breast carcinoma. *Journal of Clinical Oncology*, 16(19):3660–8.

Cooper, G. and Boron, W. (1998). Effect of PCMBS on CO2 permeability of Xenopus oocytes expressing aquaporin 1 or its C189S mutant. *American Journal of Physiology - Cell Physiology*, 275:1481–1486.

Dadiani, M., Margalit, R., Sela, N., and Degani, H. (2004). High-resolution magnetic resonance imaging of disparities in the transcapillary transfer rates in orthotopically inoculated invasive breast tumors. *Cancer Research*, 64:3155–316.

Davenport, H. (1974). *The ABC of Acid-Base Chemistry.* The University of Chicago Press.

Endeward, V. (2005). Low carbon dioxide permeability of the apical epithelial membrane of guinea-pig colon. *Journal of Physiology (London)*, 567(1):253–265.

Feldman, J. and Zimmerman, L. (2001). *Introduction to Arterial Blood Gases, Part 2.* http://missinglink.ucsf.edu/lm/abg/abg2/compensation.html.

Fencl, V., Vale, J., and Broch, J. (1969). Respiration and cerebral blood flow in metabolic acidosis and alkalosis in humans. *Journal of Applied Physiology*, 22(1):67–76.

Freireich, E., Gehan, E., Rall, D., and Schmidt, L. (1966). Quantitative comparison of toxicity of anticancer agents in mouse, rat, hamster, dog, monkey and man. *Cancer Chemotherapy Reports*, 50(4):219–44.

Gatenby, R., Gawlinski, E., Gmitro, A., Kaylor, B., and Gillies, R. (2006). Acid-mediated tumor invasion: a multidisciplinary study. *Cancer Research*, 66(10):5216–5223.

Gee, M., Procopio, W., Makonnen, S., Feldman, M., Yeilding, N., and Lee, W. (2003). Tumor vessel development and maturation impose limits on the effectiveness of anti-vascular therapy. *American Journal of Pathology*, 162(1):183–193.

Geers, C. and Gros, G. (2000). Carbon dioxide transport and carbonic anhydrase in blood and muscle. *Physiological Reviews*, 80:681–715.

Green, E. (1966). *Biology of the Laboratory Mouse.* Dover Publications.

Groothuis, D., Fischer, J., Lapin, G., Bigner, D., and Vick, N. (1982). Permeability of different experimental brain tumor models to horseradish peroxidase. *J Neuropath Exptl Neurology*, 41:164–185.

Gutknecht, J., Bisson, M., and Tosteson, F. (1971). Diffusion of carbon dioxide through lipid bilayer membranes: effects of carbonic anhydrase, bicarbonate, and unstirred layers. *Journal of General Physiology*, 69:779–794.

Hainsworth, R. (1986). *Acid-base balance.* Manchester University Press.

Hilvo, M., Baranauskiene, L., Salzano, A., Scaloni, A., Matulis, D., Innocenti, A., Scozzafava, A., Monti, S., DiFiore, A., DeSimone, G., Lindfors, M., Janis, J., Valjakka, J., Pastorekova, S., Pastorek, J., Kulomaa, M., Nordlund, H., Supuran, C., and Parkkila, S. (2008). Biochemical characterization of CA IX, one of the most active carbonic anhydrase isozymes. *Journal of Biological Chemistry*, 283:27799–809.

Hornick, D. (2003). *An Approach to the Analysis of Arterial Blood Gases and Acid-Base Disorders.* Virtual Hospital, University of Iowa Health Care [On-line].

Jain, R. (1987). Transport of molecules across tumor vasculature. *Cancer Metastasis Review*, 6(4):559–93.

Jain, R. (2005). Delivery of molecular and cellular medicine to tumors. *Tumor Pathophysiology and Transport Phenomena Course Notes*, page 55.

Javaheri, S., Shore, N. S., Rose, B., and Kazemi, H. (1982). Compensatory hypoventilation in metabolic alkalosis. *Chest*, 81(3):296–301.

Levine, D., Iacovitti, M., and Robertson, S. (2008). Modulation of single nephron GFR in the db/db mouse model of Type 2 Diabetes Mellitus. II. Effects. *American Jounal of Physiology Regulatory and Integrative Computational Physioly.*

Lote, C. (1999). *Principles of Renal Physiology.* Kluwer Academic Publishers.

Mackey, M. and Glass, L. (1977). Oscillation and chaos in physiological control systems. *Science*, 197(4300):287–9.

Martin, G. and Jain, R. (1994). Noninvasive measurement of interstitial pH profiles in normal and neoplastic tissue using fluorescence ratio imaging microscopy. *Cancer Research*, 54(21):5670–4.

Martin, N., Gaffney, E., Gatenby, R., Gillies, R., Robey, I., and Maini, P. (2011). A mathematical model of tumour and blood pH. *Mathematical Biosciences*, 230(1):1–11.

Meneton, P., Ichikawa, I., Inagami, T., and Schnermann, J. (2000). Renal physiology of the mouse. *American Journal of Physiology - Renal Physiology*, 278(3):F339–F351.

Missbach-Guentner, J., Dullin, C., Kimmina, S., Zientkowska, M., Domeyer-Missbach, M., Malz, C., Grabbe, E., Stühmer, W., and Alves, F. (2008). Morphologic changes of mammary carcinomas in mice over time as monitored by flat-panel detector volume computed tomography. *Neoplasia*, 10(7):663.

Mitchell, R. and Singer, M. (1965). Respiration and cerebrospinal fluid pH in metabolic acidosis and alkalosis. *Journal of Applied Physiology*, 20(5):905–911.

Monsky, W., Carreira, C., Tsuzuki, Y., and Gohongi, T. (2002). Role of host microenvironment in angiogenesis and microvascular functions in human breast cancer xenografts: Mammary fat pad versus cranial tumors. *Clinical Cancer Research*, 8(4):1008–13.

Nordfors, K., Haapasalo, J., Korja, M., Niemela, A., Laine, J., Parkkila, A., Pastorekova, S., Pastorek, J., Waheed, A., Sly, W., Parkkila, S., and Haapasalo, H. (2010). The tumour-associated carbonic anhydrases CA II, CA IX and CA XII in a group of medulloblastomas and supratentorial primitive neuroectodermal tumours: an association of CA IX with poor prognosis. *BMC Cancer*, (10):148.

Olesen, S. and Crone, C. (1983). Electrical resistance of muscle capillary endothelium. *Biophys J*, 42:31–41.

Pitts, R. (1970). *Physiology of the Kidney and Body Fluids*. Year Book Medical Publishers Inc.

Poppell, J., Vanamee, P., Roberts, K., and Randall, H. (1956). The effect of ventilatory insufficiency on respiratory compensations in metabolic acidosis and alkalosis. *Journal of Laboratory Clinical Medicine*, 47(6):885–90.

Putnam, R. and Roos, A. (1991). Which value for the first dissociation constant of carbonic acid should be used in biological work? *American Journal of Physiology - Cell Physiology*, 260:C1113–6.

Roberts, K., Poppell, J., Vanamee, P., Beals, R., and Randall, H. (1956). Evaluation of respiratory compensation in metabolic alkalosis. *Journal of Clinical Investigation*, 35(2):261–6.

Robey, I., Baggett, B., Kirkpatrick, N., Roe, D., Dosescu, J., Sloane, B., Hashim, A., Morse, D., Raghunand, N., Gatenby, R., and Gillies, R. (2009). Bicarbonate increases tumor pH and inhibits spontaneous metastases. *Cancer Research*, 69(6):2260–2268.

Singletary, S., Allred, C., Ashley, P., Bassett, L., Berry, D., Bland, K., Borgen, P., Clark, G., Edge, S., and Hayes, D. (2002). Revision of the American Joint Committee on cancer staging system for breast cancer. *Journal of Clinical Oncology*, 20:3628–36.

The Jackson Laboratory (2009). Mouse Tumor Biology Database, http://tumor.informatics.jax.org.

Vander, A. (1980). *Renal Physiology.* McGraw-Hill Book Co.

Wetterlin, S. and Pettersson, C. (1979). Determination of cardiac output in the mouse. *Research in Experimental Medicine*, 174(2):143–151.

Widdicombe, J. and Davies, A. (1983). *Respiratory Physiology.* Edward Arnold Publishers.

Yee, W. and Scarpelli, E. (1986). CO2 responsivity in the mouse measured by rebreathing. *Pflugers Archives*, 406(6):615–9.
